# Negative Correlation between Brain Glutathione Level and Negative Symptoms in Schizophrenia: A 3T ^1^H-MRS Study

**DOI:** 10.1371/journal.pone.0001944

**Published:** 2008-04-09

**Authors:** Daisuke Matsuzawa, Takayuki Obata, Yukihiko Shirayama, Hiroi Nonaka, Yoko Kanazawa, Eiji Yoshitome, Junichi Takanashi, Tsuyoshi Matsuda, Eiji Shimizu, Hiroo Ikehira, Masaomi Iyo, Kenji Hashimoto

**Affiliations:** 1 Department of Psychiatry, Chiba University Graduate School of Medicine, Chiba, Japan; 2 Department of Biophysics, Molecular Imaging Center, National Institute of Radiological Science, Chiba, Japan; 3 Department of Integrative Neurophysiology, Chiba University Graduate School of Medicine, Chiba, Japan; 4 Division of Pediatrics, Kameda Medical Center, Chiba, Japan; 5 Imaging Application Technical Center, GE Yokogawa Medical Systems Ltd., Tokyo, Japan; 6 Division of Clinical Neuroscience, Chiba University Center for Forensic Mental Health, Chiba, Japan; James Cook University, Australia

## Abstract

**Background:**

Glutathione (GSH), a major intracellular antioxidant, plays a role in NMDA receptor-mediated neurotransmission, which is involved in the pathophysiology of schizophrenia. In the present study, we aimed to investigate whether GSH levels are altered in the posterior medial frontal cortex of schizophrenic patients. Furthermore, we examined correlations between GSH levels and clinical variables in patients.

**Methods and Findings:**

Twenty schizophrenia patients and 16 age- and gender-matched normal controls were enrolled to examine the levels of GSH in the posterior medial frontal cortex by using 3T SIGNA EXCITE ^1^H-MRS with the spectral editing technique, MEGA-PRESS. Clinical variables of patients were assessed by the Global Assessment of Functioning (GAF), Scale for the Assessment of Negative Symptoms (SANS), Brief Psychiatric Rating Scale (BPRS), Drug-Induced Extra-Pyramidal Symptoms Scale (DIEPSS), and five cognitive performance tests (Word Fluency Test, Stroop Test, Trail Making Test, Wisconsin Card Sorting Test and Digit Span Distractibility Test). Levels of GSH in the posterior medial frontal cortex of schizophrenic patients were not different from those of normal controls. However, we found a significant negative correlation between GSH levels and the severity of negative symptoms (SANS total score and negative symptom subscore on BPRS) in patients. There were no correlations between brain GSH levels and scores on any cognitive performance test except Trail Making Test part A.

**Conclusion:**

These results suggest that GSH levels in the posterior medial frontal cortex may be related to negative symptoms in schizophrenic patients. Therefore, agents that increase GSH levels in the brain could be potential therapeutic drugs for negative symptoms in schizophrenia.

## Introduction

Accumulating evidence suggests that oxidative stress associated with impaired metabolism of the antioxidant glutathione (GSH) plays a key role in the pathogenesis of schizophrenia [1], [2]. First, activity of glutathione peroxidase (GSH-Px), a key antioxidant enzyme, was found to be decreased in red blood cells [3], [4] and plasma [5] of some, but not all schizophrenic patients [6], [7]. Furthermore, plasma GSH-Px levels were significantly and positively correlated with psychosis rating scores in schizophrenic patients [8]. Second, it has been reported that the activity of glutamate cysteine ligase (GCL), the rate-limiting enzyme for GSH synthesis, as well as expression of the catalytic GCL subunit (GCLL) protein in cultured skin fibroblasts from schizophrenic patients were significantly decreased compared to those in comparison subjects, and that decreased GCL activity was correlated with decreased GCLL protein expression [9]. Third, Do et al. [10] reported that levels of GSH in the cerebrospinal fluid of drug-free patients of schizophrenia were significantly decreased compared to those in normal comparisons. Furthermore, a study using postmortem brain samples demonstrated decreased levels of GSH, oxidized GSH (GSSG), GSH-Px, and GSH reductase in the caudate region of brains from schizophrenic patients, suggesting impaired GSH metabolism in schizophrenic brains [11]. Moreover, a 1.5T ^1^H-magnetic resonance spectroscopy (MRS) study with double quantum coherence technique demonstrated significant reduction (52 %) in GSH levels in the medial frontal cortex of schizophrenic patients compared to comparisons [10]. However, Terpstra et al. [12] reported that levels of GSH in the anterior cingulated cortex, measured by 4T ^1^H-MRS with MEGA-PRESS (MEscher-GArwood-Point RESolved Spectroscopy) sequence, did not differ in schizophrenic patients and comparisons. Fourth, several genes involved in GSH metabolism have been shown as potential candidate genes for schizophrenia. Association of the glutathione-S-transferase (GST) M1 gene was shown in schizophrenic subgroups in Japanese [13] and Korean populations [14]. Recently, Tosic et al. [15] reported that the levels of mRNA for GCLM and glutathione synthetase, which are responsible for GSH synthesis, were significantly decreased in the fibroblasts of schizophrenic patients in a Swiss population. Subsequently, they reported the GCLM gene as a susceptibility gene for schizophrenia in Swiss and Danish populations [9], [15]. Taken together, these findings provide genetic and functional evidence that an impaired capacity to synthesize GSH under conditions of oxidative stress is a vulnerability factor for schizophrenia.

GSH plays a major role in the modulation of redox-sensitive sites on the N-methyl-D-aspartate (NMDA) receptors [16]–[18], which are implicated in the pathophysiology of schizophrenia [19]–[23]. Considering the NMDA receptor hypofunction hypothesis for schizophrenia [19]–[23], it is of great interest to study whether levels of GSH are altered in the brains of schizophrenic patients. In the present study, we aimed to investigate whether GSH levels are altered in the posterior medial frontal cortex of schizophrenic patients. Furthermore, we examined the correlations between GSH levels and clinical features including the severity of clinical symptoms (positive symptoms, negative symptoms and cognitive deficits). In addition, we performed genetic analysis for the genes involved in GSH metabolism: namely, GCLM, glutathione peroxidase 1 (GPX1), and several classes (GSTM1, GSTO1, GSTP1, GSTT1 and GSTT2) of glutathione-S-transferase (GST).

## Materials and Methods

### Subjects

This research was performed under approval of the ethics committee of Chiba University Graduate School of Medicine and National Institute of Radiological Science. The experiments were thoroughly explained to the subjects, and written informed consent was obtained from all. Twenty schizophrenic patients and 16 age- and gender- matched normal controls with no past history of psychotic disorders or drug dependence were enrolled in the study. Characteristics of subjects are shown in **Table 1**. Due to a few highly educated comparisons, the extent of education and estimated IQ were significantly different between the two groups, but the estimated IQ of all patients was within the normal range. All patients were outpatients meeting the DSM-IV criteria for schizophrenia [24] and having no other psychiatric disorders. All patients were taking second-generation neuroleptics: i.e., risperidone (2–12 mg/day, n = 9), olanzapine (5–20 mg/day, n = 5), aripirprazole (6–12 mg/day, n = 4), quetiapine (500 mg/day, n = 1) or perospirone (48 mg/day, n = 1), with no change in their medication for the past month. Of the patients, twelve were diagnosed as residual type and eight were as paranoid type.

**Table 1:** Characteristics and clinical variables of subjects enrolled in this study

Variable	Controls (n = 16)	Schizophrenia (n = 20)	P values
Sex, Male/Female	12/4	12/8	0.481 a)
Age (year)	30.0±7.2 (21–41)	30.7±5.8 (20–39)	0.581 b)
Education (year)	15.2±2.9 (12–21)	13.5±1.7 (12–16)	0.04 b)
Estimated IQ*1	107.4±17.3 (90–128)	98.6±10.9 (80–114)	0.03 b)
age at onset of illness (year)		23.6±5.5 (11–31)	
Duration of illness (year)		7.30±5.2 (1–21)	
GAF scale		51.5±11.5 (29–71)	
Amount of medication*2		283.1±216(80–667)	
BPRS score		26.2±8.6 (13–43)	
BPRS positive score		12.2±5.7 (4–24)	
BPRS negative score		6.1±2.9 (2–12)	
SANS score		76.9±12.9 (60–103)	
DIEPSS score		0.41±0.15 (0.11–0.78)	
GSH (mM)	0.928±0.24 (0.608–1.465)	0.808±0.26 (0.432–1.250)	0.166 b)

All values are shown as mean±SD (range).

a) Chi-squire test,

b) Student t-test.

*1 : Short form version of Wechsler Adult Intelligence Scale, Revised (WAIS-R)

*2 : Chlorpromazine equivalent (mg)

GAF: Global Assessment of Functioning, BPRS: Brief Psychiatric Rating Scale, SANS: Scale for the Assessment of Negative Symptoms, DIEPSS: Drug Induced Extra-Pyramidal Symptoms Scale

### ^1^H-MRS measurement and data analysis

All data were acquired using the 3T SIGNA EXCITE (GE) with a standard quadrature coil. GSH spectra were acquired by the MEGA-PRESS sequence [25]. A GSH peak at chemical shift 2.95 ppm originating from cysteinyl β-CH_2_ was observed by editing pulse at 4.95 ppm α-CH resonance line J-coupled to the observed spins. Acquisition parameters for the measurement were as follows: echo time (TE) = 94 ms, repetition time (TR) = 1500 ms, number of excitations (NEX) = 512, band width 2.5 kHz, data point 4096. TE and TR were set experimentally as optimum for our system after confirming the GSH signal changes to be within a certain range (TE: 62–101 ms, TR: 1077 ms–12000 ms) with both phantom solutions and human subjects. The short TR enabled us to increase NEX and obtain a satisfying signal/noise (S/N) ratio in the human brains. For the quantification of GSH, we prepared eight phantom solutions containing different concentration of GSH (0.3–30.0 mM) with N-acetyl aspartate (NAA, 10 mM) and creatine (8 mM) to get the reference spectra. During the phantom data acquisition, the solutions were kept at 37±0.6°C.

**Figure 1:**
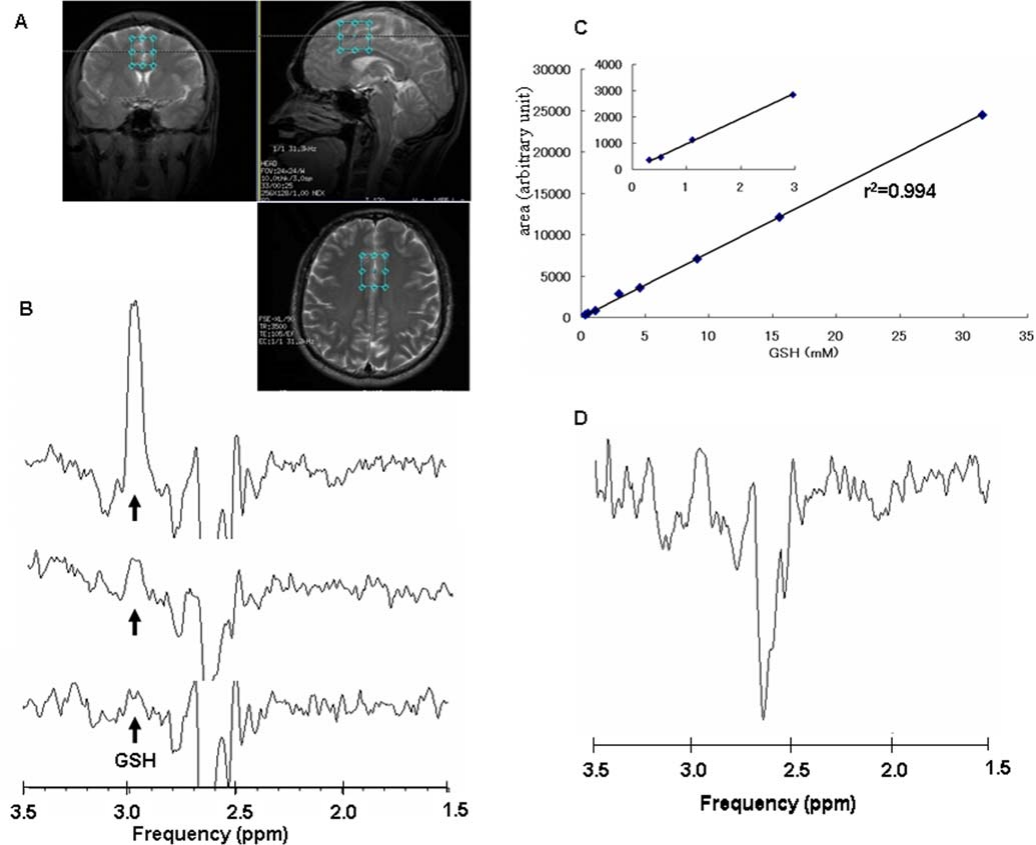
Proton MRS of GSH. (A): T2-weighed magnetic resonance imaging of the targeted region. The blue boxes show the voxel size (28 x 22 x 30 mm) in the posterior medial frontal cortex of a human brain. (B): representative data of reference phantom spectra of GSH (0.5, 1.0, 3.0 mM). Note that the GSH signal increases according to the phantom concentration. (C): Quantification of GSH. Plots showing a linear correlation (r2 = 0.994) between the GSH signal area at 2.95 ppm and the concentration of GSH. (D): Representative data of GSH signals of the posterior medial frontal cortex of a human subject. The GSH level was calculated as 0.735 mM by applying the linear concentration curve on (C).

For the acquisition of human spectra, an 18.6-ml (28×30×22 mm) volume of interest (VOI) was placed on the posterior medial frontal cortex under the guidance of T_2_-weighed images (**Figure 1A**). The posterior medial frontal cortex was selected since reduction in the GSH levels in this region of schizophrenic patients has been reported previously [10]. To minimize variation in the positioning of the head, subjects were positioned by the same investigator. The overall examination time was 1 hour or less.

For all data acquisition, high-order shim followed by automatic local shim adjustment was used and repeated until the half linewidth was accomplished under 3 Hz (phantom) or 8 Hz (human). The raw data of both phantom solutions and human subjects were processed on GE analysis software (GE Medical Systems, Milwaukee, WI). Fourier transform was done with an exponential weighting function of 2 Hz. The area of the GSH signal was measured on Image J (http://rsb.info.nih.gov/ij/) software.

### Evaluation of clinical variables

The Scale for the Assessment of Negative Symptoms (SANS) and Brief Psychiatry Rating Scale (BPRS) were used to evaluate the severity of negative symptoms and psychotic symptoms (positive and negative symptoms), respectively. The Drug-Induced Extrapyramidal Symptoms Scale (DIEPSS) was used to evaluate and exclude the effects of drug-induced extrapyramidal symptoms which could affect the severity of symptoms in schizophrenic patients. Functional disability was assessed using the Global Assessment of Functioning (GAF) scale.

### Cognitive function tests

Several cognitive function tests were used. In the Word Fluency Task (letter, category), subjects were given an initial letter (letter fluency task) or a certain category (category fluency task) as a cue [26]. Both tasks consisted of three trials, and the number of words produced in one minute for each trial was recorded for evaluation. In the Stroop Test, a list of twenty four colored dots (D), a baseline test, and 24 colored words incongruent with the color (C) were used. The difference between the reaction time (C-D) was assessed [27]. In the Wisconsin Card Sorting Test (WCST), subjects were instructed to sort cards according to a rule (color, shape, or number). The numbers of achieved categories and perseverative errors were assessed [28]. In the Trail-Making Test part (TMT) A, subjects drew lines as quickly as possible to connect 25 consecutively numbered circles. In the TMT part B, subjects connected 25 consecutively numbered and lettered circles by alternating between the two sequences. The time taken to complete each part of the test was recorded in seconds [29]. In the Digit Span Distractibility Test (DSDT), subjects were asked to remember a tape-recorded string of digits read by a female voice while ignoring the digits read by a male voice (distracter) [30].The percentages of digits correctly recalled in conditions with and without distracters were assessed separately.

### Genotyping

Genetic analysis for the genes involved in GSH metabolism-GCLM, glutathione peroxidase 1 (GPX1), and several classes of glutathione-S-transferase (GSTM1, GSTO1, GSTP1, GSTT1 and GSTT2)-as performed by the methods described previously [15], [31]–[33].

### Statistical analysis

All calculations were performed with SPSS software (SPSS version 12.0J, Tokyo, Japan). Student's t-test (unpaired) was employed for the comparison of GSH levels between schizophrenic patients and normal control subjects and of the scores of the cognitive function tests between the two groups. For the genotyping results, the differences between patients and controls were evaluated by Fisher's exact test. Pearson's correlation coefficients were examined to identify any correlations of GSH levels with the clinical severity (BPRS, SANS, and DIEPSS) of schizophrenic patients and with the scores of cognitive function tests of all subjects. A value of p<0.05 was used as the standard for statistical significance in all analyses.

## Results

### GSH concentration between schizophrenic patients and healthy comparisons

We used eight phantom solutions of different GSH concentrations (0.3–30 mM) to acquire reference spectra for quantification. As shown in **Figure 1B**, acquired GSH phantom spectra clearly increased their areas at chemical shift 2.95 ppm in a concentration-dependent manner. In **Figure 1C**, plots show a linear correlation (r^2^ = 0.994) between the GSH signal area and the GSH concentration. The areas of GSH spectra acquired from human subjects *in vivo* were applied to the linear concentration curve for quantification (**Figure 1D**). As shown in **Table 1**, GSH concentration (0.808±0.26 mM (mean±SD)) in the posterior medial frontal cortex of schizophrenic patients (n = 20) did not differ (t = 1.416, df = 34, p = 0.166) from that (0.928±0.24 mM (mean±SD)) of age- and gender-matched normal healthy controls (n = 16)(**Table 1**). Furthermore, there were no correlations between GSH levels and clinical variables (age, education, estimated IQ, age at onset of illness, duration of illness, GAF, and amount of medication) in the subjects.

### Correlation between GSH concentration and clinical variables

**Figure 2:**
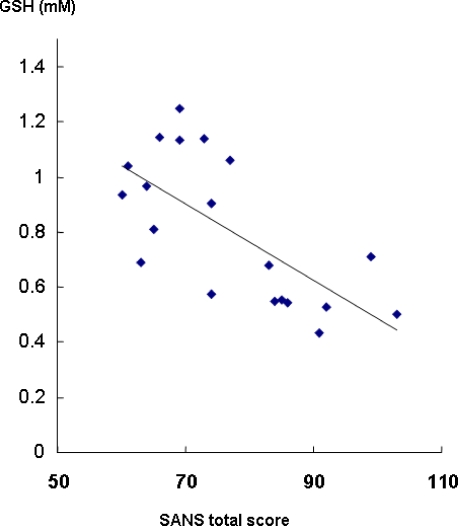
Correlation between GSH levels and the severity of negative symptoms in schizophrenia. There was a significant negative correlation (r = −068, p<0.001) between GSH levels and SANS total scores of schizophrenic patients (n = 20).

We examined the correlation between GSH level and the severity of clinical symptoms (scores of SANS, BPRS and DIEPSS) in the schizophrenic patients (n = 20). Interestingly, there was a significant negative correlation (r = −0.68, p<0.001) between GSH level and SANS total score in schizophrenic patients (**Figure 2**). Of five subscale-symptom groups in SANS, significant negative correlations with GSH level were detected in four subscales (S1: affective flattering-blunting (r = −0.57, p = 0.009), S2: alogia (r = −0.67, p = 0.001), S3: avolition-apathy (r = −0.52, p = 0.02), S4: anhedonia-asociality (r = −0.62, p = 0.004)), but not in attention impairment (r = −0.27, p = 0.252). Furthermore, we also found a significant correlation (r = −0.60, p = 0.005) between GSH levels and the negative symptom subscore on BPRS. However, there were no significant correlations between GSH level and BPRS total score (r = −0.41, p = 0.076), BPRS positive symptom score (r = −0.43, p = 0.059) and DIEPSS score (r = −0.32, p = 0.167). Because these correlations might have been affected by medication, we controlled for the doses of antipsychotics using partial correlation coefficients. Even when the administered antipsychotics (chlorpromazine equivalents) were adjusted for using partial correlation coefficients, the relationships between GSH level and SANS score (partial correlation coefficient = −0.60, p = 0.007) or BPRS negative score (partial correlation coefficient = −0.52, p = 0.02) remained significant.

### Correlation between GSH concentration and cognitive functions

As shown in **Table 2**, significant differences were observed between schizophrenia patients and normal controls in all cognitive function tests: Word Fluency (letters: t = 4.67, df = 34, p<0.001; category: t = 3.57, df = 34, p<0.01), Stroop Task (t = −3.47, df = 34, p<0.01), WCST (category: t = 3.95, df = 34, p<0.001; perseverative error : t = −4.61, df = 34, p<0.001), Trail Making Test (TMT-A: t = −3.21, df = 34, p<0.001; TMT-B: t = −3.43, df = 34, p<0.01; TMT-B-A; t = −2.17, df = 34, p = 0.03), and DSDT (without distracter: t = 1.35, df = 34, p = 0.18; with distracter; t = 3.23, df = 34, p<0.01).

**Table 2:** Performance on cognitive function tests and their correlations with GSH level

Cognitive function test	mean scores±SD		Coefficients with GSH level (r)a)
	Control subjects (n = 16)	Schizophrenia (n = 20)	
Word Fluency (letter)	41.3±8.8	28.4±7.8***	0.15
Word Fluency (category)	48.9±8.4	39.8±6.9**	0.21
Stroop test (C-D, sec)	5.8±3.9	12.1±6.4**	−0.05
WCST (category)	5.1±1.9	2.7±1.8**	0.01
WCST (perserverative error)	2.1±2.5	11.5±8.4***	−0.23
TMT-A (sec)	21.8±6.7	32.0±8.4**	−0.36*
TMT-B (sec)	48.4±18.2	80.5±32.1**	−0.14
TMT B-A (sec)	26.6±13.5	53.6±51.3*	0.06
DSDT (without distractor)	87.9±12.8	80.3±20.8	0.18
DSDT (with distractor)	93.0±7.2	74.4±22.1**	0.31

* P <0.05

** P <0.01

*** P <0.0001 (vs.Control)

a) Pearson's coefficients between GSH level in all subjects (n = 36). ^*^P <0.05

WCST : Wisconsin Card Sorting Test, TMT : Trail Making Test, DSDT : Digit Span Distractibility Test

Then, we examined the correlations between GSH levels and the scores of cognitive function tests. We found a significant negative correlation (r = −0.36, p = 0.03) between GSH level and TMT-A scores in all subjects (n = 36)(**Table 2**). There were no correlations between GSH levels and the scores of other cognitive function tests (**Table 2**).

### Correlations between GSH concentration and the genotypes of enzymes related with GSH metabolism

There was a significantly (p = 0.017) different genotype distribution for the GSTT2 gene between schizophrenic patients and healthy controls. No different distribution was observed in other genes (**Table S1**). Then, we investigated whether or not these genotypes affected GSH levels in the posterior medial frontal cortex. There were no significant differences in GSH levels relevant to those genotypes. However, we found a difference in GSH levels between patients (n = 15) and normal controls (n = 5) in subjects with the G/G genotype of the GSTT2 (Met139Ile) gene, although the difference only showed a trend toward statistical significance (p = 0.058) (**Figure S1**). We also found a difference in GSH levels between patients (n = 13) and normal controls (n = 11) in subjects with the C/C genotype of the GCLM (ss60297536) gene; again, the differences only showed a trend toward statistical significance (p = 0.099) (**Figure S1**).

## Discussion

The major finding of this study was that GSH levels in the posterior medial frontal cortex of schizophrenic patients were significantly correlated with the severity of their negative symptoms. To the best of our knowledge, this is the first report demonstrating the negative correlation between brain levels of GSH and the severity of negative symptoms in schizophrenia.

The measurement of brain GSH levels by ^1^H-MRS has been elusive up until now because GSH exists at a relatively low concentration and the cysteinyl β-CH_2_ signal of GSH at 2.95ppm overlaps with other resonances such as those of aspartate, γ-aminobutylic acid (GABA), and especially creatine, with its high concentration in human brain. The MEGA-PRESS sequence is able to highlight the GSH signal by adding two editing pulses with a normal PRESS sequence. Sufficient GSH signal was obtained by setting an optimum condition with repeated preliminary measurements using both phantom solutions and human subjects, and the shorter TR than in previous studies [12], [25], [34] enabled us to increase the number of scans within the short examination time.

In this study, we found no alteration in GSH concentrations in the brains of schizophrenic patients, which was consistent with a previous report using the MEGA-PRESS sequence [12], but not a previous report using a double quantum coherence filter technique [10]. The reasons underlying this discrepancy are currently unclear. One possibility may be due to the difference of technique (MEGA-PRESS sequence vs. a double quantum coherence filter) for GSH measurement. Another possibility may be due to medication. The patients enrolled in the study of Do et al. [10] were first-episode patients whereas those in the present study and that of Terpstra et al. [12] were medicated. However, in this study, we found no effect of medication on GSH levels in schizophrenic patients. Therefore, it is unlikely that medication contributes to this discrepancy, although further study is necessary.

The present finding suggests that increasing the brain levels of GSH should be considered a potential therapeutic approach for negative symptoms in schizophrenia. It is well known that oral administration of GSH does not result in its effective increase in the brain because of its poor penetration through the blood-brain barrier, indicating that GSH is not a suitable agent for treating neuropsychiatric diseases such as schizophrenia. The antioxidant N-acetyl-L-cysteine (NAC) has been widely used as a donor of cysteine, the limiting precursor in the synthesis of GSH, and NAC has a good penetration through the blood-brain barrier. Recently, Lavoie et al. [35] reported that treatment of schizophrenic patients with NAC significantly improved impaired mismatch negativity, which is an auditory evoked potential component related to NMDA receptor function [36]. Furthermore, a multi-center double-blinded trial of NAC showed improvement of negative symptoms on the Positive and Negative Symptoms Scale after 6 months of treatment with NAC ([36], Berk et al, unpublished work). Interestingly, it has been reported that GSH-deficient mice showed enhanced dopamine neurotransmission, altered serotonin function, and augmented locomotor responses to low doses of the NMDA receptor antagonist phencyclidine, suggesting that the GSH deficiency produced alterations in monoaminergic function and behavior in mice relevant to schizophrenia [37]. Moreover, we reported that NAC could attenuate behavioral changes and neurotoxicity in rodents and non-human primates after repeated administration of the psychostimulant methamphetamine [38], [39]. Taken together, the findings suggest that NAC has potential as a therapeutic drug for negative symptoms in schizophrenia.

In this study, we found a weak negative correlation between GSH levels in the posterior medial frontal cortex and TMT-A scores. There was also a positive correlation (r = 0.47, p = 0.024) between TMT-A scores and SANS total scores in schizophrenic patients. The posterior medial frontal cortex can be divided functionally into two parts: an upper half including Brodman areas 8 and 9 and a lower half including part of the anterior cingulate cortex, Brodman areas 24 and 32 [40]. Both parts are shown to play a role in self monitoring and control of action demanded in the context of social cognitive processes [40]. The relation between GSH level and cognitive symptoms might be assessed in more detail by setting smaller and more specific VOI in the brain, although it is currently difficult to get sufficient GSH signal with smaller VOI. Nonetheless, it seems that GSH levels in the posterior medial frontal cortex may be associated with cognitive impairment as well as negative symptoms in schizophrenia. Therefore, GSH levels in the posterior medial frontal cortex may be a predictive biological factor for the severity of cognitive impairment and negative symptoms in schizophrenia.

In this study, GSH levels were not affected by the genotypes of several genes related to GSH metabolism. The genotype distribution of GSTT2 was significantly (p = 0.017) different between patients (n = 20) and normal controls (n = 16), but this was considered to be a type I error due to the small sample size, as our study using a larger sample size (over 200 of both groups) revealed no significant difference (Matsuzawa et al, submitted). Interestingly, we found that brain GSH levels in patients with the C/C genotype of the GCLM (ss60297536) gene were lower than those of controls with the C/C genotype of the GCLM (ss60297536) gene although the differences only showed a trend toward statistical significance (p = 0.099). Further study using a large sample will be necessary to study the relationship between GCLM gene polymorphism and GSH levels in schizophrenic patients.

In conclusion, the present study suggests a negative correlation between GSH levels in the posterior medial frontal cortex and the severity of negative symptoms in schizophrenia. Therefore, agents (e.g., NAC) that can increase brain GSH levels should be considered potential therapeutic drugs for negative symptoms in schizophrenia.

## Supporting Information

Figure S1 GSH levels and the relevance with polymorphisms of GCLM and GSTT2 gene. The plots show GSH levels of controls and patients with each genotype of GCLM-588 (left) and GSTT2 (right). The bars represent mean GSH level ± standard deviation (mM).
(0.20 MB TIF)

Table S1 (0.05 MB DOC)

## References

[1] Mahadik SP, Mukherjee S (1996). Free radical pathology and antioxidant defense in schizophrenia: A review.. Schizophr Res.

[2] Yao JK, Reddy RD, van Kammen DP (2001). Oxidative damage and schizophrenia: An overview of the evidence and its therapeutic implications.. CNS Drugs.

[3] Abdalla DS, Monteiro HP, Oliveira JA, Bechara EJ (1986). Activities of superoxide dismutase and glutathione peroxidase in schizophrenic and manic-depressive patients.. Clin Chem.

[4] Ben Othmen L, Mechri A, Fendri C, Bost M, Chazot G (2008). Altered antioxidant defense system in clinically stable patients with schizophrenia and their unafected siblings.. Progress in Neuro-Psychopharmacology & Biological Psychiatry.

[5] Zhang XY, Tan YL, Zhou DF, Cao LY, Wu GY (2007). Disrupted antioxidant enzyme activity and elevated lipid peroxidation products in schizophrenic patients with tardive dyskinesia.. J Clin Psychiatry.

[6] Reddy R, Sahebarao MP, Mukherjee S, Murthy JN (1991). Enzymes of the antioxidant defense system in chronic schizophrenic patients.. Biol Psychiatry.

[7] Akyol O, Herken H, Uz E, Fadillioglu E, Unal S (2002). The indices of endogenous oxidative and antioxidative processes in plasma from schizophrenic patients. The possible role of oxidant/antioxidant imbalance.. Prog Neuropsychopharmacol Biol Psychiatry.

[8] Yao JK, Reddy RD, van Kammen DP (1999). Human plasma glutathione peroxidase and symptom severity in schizophrenia.. Biol Psychiatry.

[9] Gysin R, Kraftsik R, Sandell J, Bovet P, Chappuis C (2007). Impaired glutathione synthesis in schizophrenia: Convergent genetic and functional evidence.. Proc Natl Acad Sci USA.

[10] Do KQ, Trabesinger AH, Kirsten-Kruger M, Lauer CJ, Dydak U (2000). Schizophrenia: Glutathione deficit in cerebrospinal fluid and prefrontal cortex *in vivo*.. Eur J Neurosci.

[11] Yao JK, Leonard S, Reddy R (2006). Altered glutathione redox state in schizophrenia.. Dis Markers.

[12] Terpstra M, Vaughan TJ, Ugurbil K, Lim KO, Schulz SC (2005). Validation of glutathione quantitation from STEAM spectra against edited 1H NMR spectroscopy at 4T: Application to schizophrenia.. MAGMA.

[13] Harada S, Tachikawa H, Kawanishi Y (2001). Glutathione S-transferase M1 gene deletion may be associated with susceptibility to certain forms of schizophrenia.. Biochem Biophys Res Commun.

[14] Pae CU, Yu HS, Kim JJ, Kim W, Lee CU (2004). Glutathione S-transferase M1 polymorphism may contribute to schizophrenia in the korean population.. Psychiatr Genet.

[15] Tosic M, Ott J, Barral S, Bovet P, Deppen P (2006). Schizophrenia and oxidative stress: Glutamate cysteine ligase modifier as a susceptibility gene.. Am J Hum Genet.

[16] Sucher NJ, Lipton SA (1991). Redox modulatory site of the NMDA receptor-channel complex: Regulation by oxidized glutathione.. J Neurosci Res.

[17] Kohr G, Eckardt S, Luddens H, Monyer H, Seeburg PH (1994). NMDA receptor channels: Subunit-specific potentiation by reducing agents.. Neuron.

[18] Varga V, Jenei Z, Janaky R, Saransaari P, Oja SS (1997). Glutathione is an endogenous ligand of rat brain N-methyl-D-aspartate (NMDA) and 2-amino-3-hydroxy-5-methyl-4-isoxazolepropionate (AMPA) receptors.. Neurochem Res.

[19] Javitt DC, Zukin SR (1991). Recent advances in the phencyclidine model of schizophrenia.. Am J Psychiatry.

[20] Tamminga CA (1998). Schizophrenia and glutamatergic transmission.. Crit Rev Neurobiol.

[21] Goff DC, Coyle JT (2001). The emerging role of glutamate in the pathophysiology and treatment of schizophrenia.. Am J Psychiatry.

[22] Hashimoto K, Fukushima T, Shimizu E, Komatsu N, Watanabe H (2003). Decreased serum levels of D-serine in patients with schizophrenia: Evidence in support of the N-methyl-D-aspartate receptor hypofunction hypothesis of schizophrenia.. Arch Gen Psychiatry.

[23] Hashimoto K, Okamura N, Shimizu E, Iyo M (2003). Glutamate hypothesis of schizophrenia and approach for possible therapeutic drugs.. Curr Med Chem-CNS Agents.

[24] American Psychiatric Association: Diagnostic and Statistical Manual of Mental Disorders (4th Ed) (1994).

[25] Terpstra M, Henry PG, Gruetter R (2003). Measurement of reduced glutathione (GSH) in human brain using LCModel analysis of difference-edited spectra.. Magn Reson Med.

[26] Sumiyoshi C, Sumiyoshi T, Nohara S, Yamashita I, Matsui M (2005). Disorganization of semantic memory underlies alogia in schizophrenia: An analysis of verbal fluency performance in japanese subjects.. Schizophr Res.

[27] Carter CS, Mintun M, Cohen JD (1995). Interference and facilitation effects during selective attention: An H215O PET study of stroop task performance.. Neuroimage.

[28] Shad MU, Tamminga CA, Cullum M, Haas GL, Keshavan MS (2006). Insight and frontal cortical function in schizophrenia: A review.. Schizophr Res.

[29] Reitan R, Wolfson D (1985). The halstead-reitan neuropsychological test battery..

[30] Wilder-Willis KE, Sax KW, Rosenberg HL, Fleck DE, Shear PK (2001). Persistent attentional dysfunction in remitted bipolar disorder.. Bipolar Disord.

[31] Matsuzawa D, Hashimoto K, Shimizu E, Fujisaki M, Iyo M (2005). Functional polymorphism of the glutathione peroxidase 1 gene is associated with personality traits in healthy subjects.. Neuropsychobiology.

[32] Hashimoto T, Hashimoto K, Matsuzawa D, Shimizu E, Sekine Y (2005). A functional glutathione S-transferase P1 gene polymorphism is associated with methamphetamine-induced psychosis in japanese population.. Am J Med Genet B Neuropsychiatr Genet.

[33] Hashimoto T, Hashimoto K, Miyatake R, Matsuzawa D, Sekine Y (2008). Association study between polymorphisms in glutathione-related genes and methamphetamine use disorder in a japanese population.. Am J Med Genet B Neuropsychiatr Genet in press.

[34] Satoh T, Yoshioka Y (2006). Contribution of reduced and oxidized glutathione to signals detected by magnetic resonance spectroscopy as indicators of local brain redox state.. Neurosci Res.

[35] Lavoie S, Murray MM, Deppen P, Knyazeva MG, Berk M (2007). Glutathione precursor, N-acetyl-cysteine, improves mismatch negativity in schizophrenia patients.. Neuropsychopharmacology in press.

[36] Javitt DC, Steinschneider M, Schroeder CE, Arezzo JC (1996). Role of cortical N-methyl-D-aspartate receptors in auditory sensory memory and mismatch negativity generation: Implications for schizophrenia.. Proc Natl Acad Sci USA.

[37] Jacobsen JP, Rodriguiz RM, Mork A, Wetsel WC (2005). Monoaminergic dysregulation in glutathione-deficient mice: Possible relevance to schizophrenia?. Neuroscience.

[38] Fukami G, Hashimoto K, Koike K, Okamura N, Shimizu E (2004). Effect of antioxidant N-acetyl-L-cysteine on behavioral changes and neurotoxicity in rats after administration of methamphetamine.. Brain Res.

[39] Hashimoto K, Tsukada H, Nishiyama S, Fukumoto D, Kakiuchi T (2004). Protective effects of N-acetyl-L-cysteine on the reduction of dopamine transporters in the striatum of monkeys treated with methamphetamine.. Neuropsychopharmacology.

[40] Amodio DM, Frith CD (2006). Meeting of minds: The medial frontal cortex and social cognition.. Nat Rev Neurosci.

