# Supplementary material for: Negative Correlation between Brain Glutathione Level and Negative Symptoms in Schizophrenia: A 3T 1H-MRS Study
Source: PLoS One. 2008 Apr 9;3(4):e1944. doi: 10.1371/journal.pone.0001944 (PMC2275307; doi:10.1371/journal.pone.0001944)
Supplement: Table S1 — (0.05 MB DOC) [file pone.0001944.s002.doc]

| | **Table S1. Genotype frequencies in schizophrenic patients and controls** | | | | | | | | | --- | --- | --- | --- | --- | --- | --- | --- | |  |  |  | Frequency (%) | | | | | | Gene name | Genotype |  | Controls |  | Patients |  | p^*1^ | | GCLM-588 | C/C |  | 11 (68.8%) |  | 13 (65.0%) |  |  | | ss60197536 | C/T, T/T |  | 5 (32.2%) |  | 7 (35.0%) |  | 1 | | GCLM | G/G |  | 8 (50.0%) |  | 10 (50.0%) |  |  | | rs230102 | G/A, A/A |  | 8 (50.0%) |  | 10 (50.0%) |  | 1 | | GSTM1 | Present |  | 5 (31.3%) |  | 11 (55.0%) |  |  | |  | Null |  | 11(68.8%) |  | 9 (45.0%) |  | 0.191 | | GSTT1 | Present |  | 11 (68.8%) |  | 11 (55.0%) |  |  | |  | Null |  | 5 (31.3%) |  | 9 (45.0%) |  | 0.501 | | GSTT2 | Met/Met |  | 5 (31.3%) |  | 15 (75.0%) |  |  | | (Met139Ile) | Met/Ile, Ile/Ile |  | 11 (68.7%) |  | 5 (25.0%) |  | 0.017 | | GSTO1 | Ala/Ala |  | 13 (81.3%) |  | 16 (80.0%) |  |  | | (Ala140Asp) | Ala/Asp, Asp/Asp |  | 3 (18.7%) |  | 4 (20.0%) |  | 1 | | GSTP1 | Ile/Ile |  | 12 (75.0%) |  | 16 (80.0%) |  |  | | (Ile105Val) | Ile/Val, Val/Val |  | 4 (25.0%) |  | 4 (20.0%) |  | 1 | | GPX1 | Pro/Pro |  | 13 (81.3%) |  | 18 (90.0%) |  |  | | (Pro198Leu) | Pro/Leu, Leu/Leu |  | 3 (18.7%) |  | 2 (10.0%) |  | 0.637 | | *1 Fischer's exact test, schizophrenia vs. controls | | | | | | | | |
| --- | --- | --- | --- | --- | --- | --- | --- | --- | --- | --- | --- | --- | --- | --- | --- | --- | --- | --- | --- | --- | --- | --- | --- | --- | --- | --- | --- | --- | --- | --- | --- | --- | --- | --- | --- | --- | --- | --- | --- | --- | --- | --- | --- | --- | --- | --- | --- | --- | --- | --- | --- | --- | --- | --- | --- | --- | --- | --- | --- | --- | --- | --- | --- | --- | --- | --- | --- | --- | --- | --- | --- | --- | --- | --- | --- | --- | --- | --- | --- | --- | --- | --- | --- | --- | --- | --- | --- | --- | --- | --- | --- | --- | --- | --- | --- | --- | --- | --- | --- | --- | --- | --- | --- | --- | --- | --- | --- | --- | --- | --- | --- | --- | --- | --- | --- | --- | --- | --- | --- | --- | --- | --- | --- | --- | --- | --- | --- | --- | --- | --- | --- | --- | --- | --- | --- | --- | --- | --- | --- | --- | --- | --- | --- | --- | --- | --- | --- | --- | --- | --- | --- | --- | --- | --- | --- | --- | --- | --- | --- | --- |
